# A research perspective on sphingolipid metabolism and myalgic encephalomyelitis/chronic fatigue syndrome

**DOI:** 10.4103/NRR.NRR-D-24-01506

**Published:** 2025-03-25

**Authors:** Junhua Xiao

**Affiliations:** Department of Biomedical, Health and Exercise Sciences, School of Health Sciences, Swinburne University of Technology, Hawthorn, VIC, Australia

**Myalgic encephalomyelitis/chronic fatigue syndrome–an insidious disease:** The recent COVID-19 pandemic has brought substantial attention to the overlapping symptoms between long COVID and myalgic encephalomyelitis/chronic fatigue syndrome (ME/CFS), a chronic and poorly understood neurological disorder (Shankar et al., 2024). The prevalence of ME/CFS is estimated to be over 2 million in the United States (No authors listed, 2015). People with ME/CFS often report prolonged fatigue, chronic or intermittent pain syndromes, autonomic abnormalities, post-exertional malaise, cognitive impairment, and reduced quality of life over an enduring period, many of which do not fully recover from this clinical condition (No authors listed, 2015). Furthermore, the condition is marked by considerable heterogeneity in its clinical definitions and the absence of a definitive diagnostic test, complicating both diagnosis and treatment. Because physicians often struggle to recognize the condition of ME/CFS and form a diagnosis, many individuals are never diagnosed hence the actual number would be substantially higher, posing additional complexity in developing evidence-based therapies for ME/CFS.

Current therapies for ME/CFS are limited to alleviating symptoms or psychological sequelae. Supplementary treatments that target oxidative stress have failed to achieve the desired outcome in ME/CFS (Morris et al., 2019). Cognitive behavioral therapy and graded exercise are of modest benefit for only some ME/CFS patients, and many sufferers report aggravation of symptoms of fatigue with exercise (Germain et al., 2022). Unfortunately, no intervention has been proven effective in ME/CFS. A more complete understanding of the underlying mechanistic basis will help develop evidence-based therapeutic strategies for effectively managing ME/CFS.

**Figure 1:**
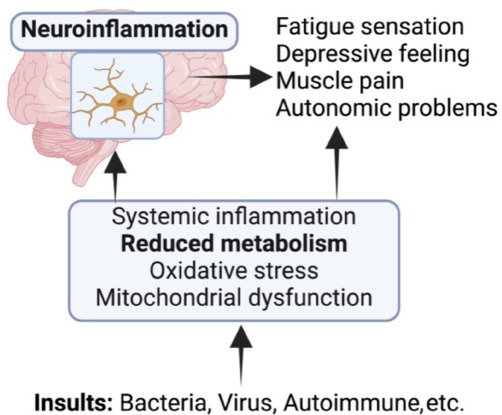
Schematic diagram illustrating the development of myalgic encephalomyelitis/chronic fatigue syndrome. Myalgic encephalomyelitis/chronic fatigue syndrome (ME/CFS) involves an interplay between the immune and nervous systems involving systemic and central inflammation, mitochondrial dysfunction, oxidative stress, and altered metabolism. While the etiology is currently unknown, ME/CFS is believed to be associated with various inflammatory insults, including viral or bacterial infection as well as autoimmune factors. Despite the heterogeneous triggers, the development of ME/CFS is believed to be underpinned ultimately by inflammation and reduced metabolism. Indeed, the extent of inflammation is closely associated with disease severity in people with ME/CFS. A consistent biochemical response in people with ME/CFS is reduced metabolism. Systemic and neural inflammation exerts adverse changes in the nervous system structures and function. These pathological changes together could trigger various symptoms experienced in people with ME/CFS, including fatigue, depression, pain, and problems with the autonomic nervous system. Created with BioRender.com.

**Myalgic encephalomyelitis/chronic fatigue syndrome pathophysiology–inflammation and abnormal metabolism:** Emerging evidence suggests ME/CFS involves an interplay between the immune and nervous system, involving inflammation, mitochondrial dysfunction, and oxidative stress (**Figure 1**). While the etiology is currently unknown, ME/CFS is often associated with viral or bacterial infection as well as trauma (Morris et al., 2019). In addition, a subset of patients has an autoimmune etiology. Interestingly, ME/CFS is comparable to autoimmune disease in particular multiple sclerosis, in prevalence (0.2% to 1%), sex ratio, and long-term disability. Despite heterogeneous triggers, a prevailing hypothesis is that the development of ME/CFS is ultimately underpinned by inflammation and altered metabolism (Morris et al., 2019; Germain et al., 2020; Che et al., 2022; **Figure 1**). Recent studies in mitochondria have begun to generate a scientific basis that supports a broader metabolic dysfunction over various physiological systems in ME/CFS (Morris et al., 2019). It has been well established that systemic and neural inflammation exerts adverse changes in the nervous system structures and function.

Currently, no one animal model faithfully mimics all aspects of ME/CFS pathophysiology (Morris et al., 2019), posing additional challenges to the study of ME/CFS pathophysiology and therapies. Albeit several experimental models of neuroinflammation could apply to the study of ME/CFS in a pre-clinical setting. The lipopolysaccharide model of neuroinflammation is an infection-induced model of neuroinflammation, commonly used to mimic bacterial infection-induced ME/CFS. This model mimics several pathological relevances of ME/CFS, such as fatigue, depression, and neuroinflammation (Harland et al., 2020). The major advantage of the lipopolysaccharide model is that it allows quantifiable analyses of behavioral and structural deficits. While lipopolysaccharide is a commonly used model for studying neuroinflammation due to its high reproducibility and low costs, its application is limited to studying the aspect of neuroinflammation induced by bacterial infections. The experimental autoimmune encephalomyelitis model is an autoimmune model of neuroinflammation. The experimental autoimmune encephalomyelitis displays a quantifiable functional deficit and mimics several pathological relevances of ME/CFS, such as fatigue, neuropathic pain, and neuroinflammation (Ondek et al., 2021). Its autoimmune mechanism will provide important insights into the understanding of a subset of ME/CFS patients with autoimmune etiology. Cognisant of the heterogeneous triggers in this disease, adopting different animal models of neuroinflammation with complementary features will help address questions relating to ME/CFS.

**Sphingolipid–a dominant metabolic pathway involved in myalgic encephalomyelitis/chronic fatigue syndrome:** Despite the various inflammatory insults, shifts in metabolism, particularly disrupted lipid metabolisms (Naviaux et al., 2016; Maya et al., 2023), appear to be a consistent chemical signature of ME/CFS development (**Figure 1**). In 2016, Naviaux et al. published a milestone paper revealing the metabolic features of ME/CFS, identifying sphingolipid as a dominant metabolic pathway. The metabolism of sphingolipid is consistently reduced in people with ME/CFS, whilst other common metabolites such as isoleucine, valine, and glucose remain unchanged. Abnormal sphingolipid metabolism is also observed in other diseases driven by chronic fatigue such as Q-fever fatigue syndrome (Raijmakers et al., 2020) and oxidant-mediated skeletal muscle fatigue (Ferreira et al., 2010). These studies collectively identify sphingolipid as an important chemical signature in regulating chronic fatigue ME/CFS as it not only helps develop a diagnostic test and monitor individualized treatment responses but also potentially reveals a pathological pathway underpinning fatigue development, which could be selectively targeted for therapeutic intervention.

Sphingolipids are ubiquitously expressed throughout various organ systems. A shift in its metabolism could disturb immunity and various inflammatory pathways. Sphingolipids comprise a family of bioactive lipids including sphingosine, ceramide, and sphingosine-1-phosphate (S1P) (Binish and Xiao, 2025). These sphingolipids exert many physiological roles including inflammation, mitochondrial biosynthesis, and cell survival (Binish and Xiao, 2025). Directly relevant to ME/CFS, sphingolipid metabolites such as S1P not only inhibit inflammation and enhance neuroprotection (Binish and Xiao, 2025) but also protect against fatigue development and regulate skeletal muscle metabolism (Cordeiro et al., 2019). At least half of the people with ME/CFS (both males and females) displayed a widespread decrease in plasma sphingolipids and glycosphingolipids (Naviaux et al., 2016). 20–30 molecular species of sphingolipids were decreased in people with ME/CFS, with the majority being ceramide. Despite the significance, however, it is unknown whether restoring sphingolipid metabolites could be a new strategy that can inhibit neuroinflammation and protect against the development of ME/CFS, a gap that needs to be redressed in the future. Future research could focus on determining whether selectively targeting the sphingolipid metabolic pathway is a new strategy that can effectively harness ME/CFS development. One of the underlying challenges is how to selectively modulate key metabolites of sphingolipids within an interconnected network (Binish and Xiao, 2025).

Sphingolipids are a class of membrane lipids containing a backbone of sphingoid bases, sphingosine. They function both as structural components of cell membranes and as signaling molecules, linking metabolism to signaling pathways. Recently, numerous reviews about sphingolipid metabolisms and physiology have been published. The metabolic pathway of sphingolipids is a highly coordinated system linking together various pathways and involves sequential reaction sequences in the formation of ceramide and other sphingolipids (Binish and Xiao, 2025). Sphingolipid metabolites can be produced via *de novo* or salvage pathways. Ultimately, ceramide represents a molecule central to sphingolipid metabolism and a key intermediate in the production of sphingosine and S1P (Binish and Xiao, 2025). Ceramide participates in stress-induced signaling pathways. Being in the hub of the sphingolipid network, ceramide has served as a major research focus for over 20 years, although whether it is neuroprotective or neurotoxic remains unclear, which is context dependent. While significant effort has been directed toward the implication of ceramide in cancer research, recent research has investigated the roles of ceramide in multiple pathophysiological processes including immunity, inflammation, and metabolic diseases including diabetes and muscle fatigue (Ferreira et al., 2010). For example, the treatment of skeletal muscle with a sphingomyelinase enzyme, an enzyme that hydrolyzes membrane sphingomyelin into ceramide, results in muscle weakness and promotes muscle fatigue (Ferreira et al., 2010). Future research should investigate if strategies that selectively modulate sphingolipid metabolites such as ceramide can effectively reverse muscle fatigue.

A notable feature of the sphingolipid metabolic pathway is its dynamic Interconversion as the pathway exhibits a high level of interconnectivity regulated by subsequential enzyme reactions, most of which are reversible. There is one enzyme, sphingosine 1-phosphate lyase (SPL), which acts as the gatekeeper of sphingolipid metabolic flow. It is the terminal enzyme that controls the final exit of sphingolipids through irreversibly degrading S1P. Therefore, SPL is placed in a unique position that controls sphingolipid metabolic flow and “guards” S1P levels within both circulation and tissues. Modulating SPL influences not only the amount of S1P available for signaling but also the overall homeostasis of sphingolipid metabolites. In this context, inhibiting SPL, by implication restoring the reduced sphingolipid metabolites may be a new approach to managing ME/CFS, which warrants future investigation. Indeed, this interest in identifying selective SPL inhibitors for inflammatory neurological disorders has been ongoing and reflected in the current drug development by Abbvie and Novartis. To date, however, only a limited number of SPL inhibitors have been identified and almost all of them are indirect inhibitors of SPL with poor selectivity and efficacy (George and Xiao, 2024). Moreover, none of the reported SPL inhibitors demonstrate their molecular specificity nor efficacy in ameliorating central nervous system neuroinflammation in a therapeutically relevant paradigm (George and Xiao, 2024). Therefore, with the interest in exploring new therapeutics that target sphingolipid metabolism for ME/CFS, there is a demonstrated need to develop and evaluate new SPL inhibitors with proven efficacy in ameliorating fatigue development and combating neuroinflammation, which requires future research attention.

In summary, ME/CFS is a chronic and heterogeneous condition where uncertain insults trigger wide perturbations in varying physiological systems. The inflammation and immune dysfunction developed in ME/CFS are thought to drive a shift in metabolic states, contributing to impaired energy production, autonomic dysregulation, and cognitive dysfunction. A more complete understanding of the mechanism underpinning fatigue pathophysiology is essential for developing effective therapies. Recent discoveries, such as the role of sphingolipids as a chemical signal of ME/CFS, have shed light on the significance of lipid metabolism in fatigue development. This new insight invites future research focusing on dissecting the roles of sphingolipid metabolism and its contribution to the pathophysiology of ME/CFS, paving the way for the future development of new therapies.

*This work was supported by the Judith Jane Mason and Harold Stannett Williams Memorial Foundation National Medical Program (#Mason2210) to JX*.
